# Long-Term Patient-Reported Outcomes After Ventral Stabilization of Thoracolumbar Fractures

**DOI:** 10.3390/medicina62040760

**Published:** 2026-04-15

**Authors:** Katharina Jäckle, Paul-Jonathan Roch, Friederike Eva Roch, Friederike Sophie Klockner, Lina Franziska Höller, Marc-Pascal Meier, Thelonius Hawellek, Hassan Awan Malik, Wolfgang Lehmann, Lukas Weiser

**Affiliations:** Department of Trauma Surgery, Orthopaedic Surgery and Plastic Surgery, University Medical Center Göttingen, Robert-Koch Str. 40, 37075 Göttingen, Germany

**Keywords:** clinical outcome, spine surgery, cage implantation, autologous pelvic bone graft, vertebral body replacement, thoracolumbar fracture

## Abstract

*Background and Objectives*: Ventral stabilization of thoracolumbar spine fractures can be achieved using different interbody reconstruction techniques, including titanium cages, vertebral body replacements (VBR), and autologous pelvic bone grafts (APBG). Although all approaches aim to restore anterior column stability and alignment, comparative data on long-term patient-reported outcomes remain limited. The objective of this study was to compare long-term patient-reported wellbeing following ventral stabilization using these three techniques. *Materials and Methods*: A retrospective, non-randomized single-center cohort study with prospective follow-up was analyzed. Treatment allocation was indication-based. Ninety-one patients treated between 2008 and 2018 underwent ventral stabilization using cage implantation (*n* = 12), vertebral body replacement (*n* = 45), or autologous pelvic bone grafting (*n* = 34). Clinical outcome was assessed at least 12 months postoperatively using a modified Visual Analog Scale Spine Score (VAS-Spine). Statistical analysis included linear and ordinal regression adjusted for age and sex. Potential baseline differences between groups were considered in the interpretation of the results. *Results*: Sixty-three patients (mean age 52 ± 15 years; 41% female) completed follow-up. The mean VAS-Spine score was lowest after cage implantation (2.7 ± 3.6), followed by VBR (3.9 ± 2.8) and APBG (4.9 ± 1.8; p* = 0.021). The observed difference between cage and APBG approached the minimal clinically important difference reported for VAS-based measures. Patients treated with cage implantation reported less pain during rest and activity and fewer limitations in daily life. No significant differences were observed regarding age or sex. *Conclusions*: In this observational cohort, cage implantation was associated with more favorable patient-reported outcomes compared with VBR and APBG. Autologous pelvic bone grafting was associated with worse patient-reported outcomes, potentially related to donor-site morbidity. Given the non-randomized design and potential confounding, these findings should be interpreted as associative and hypothesis-generating.

## 1. Introduction

The incidence of vertebral body fractures is highly age-dependent. They occur in approximately 13 per 100,000 individuals under 60 years of age but increase sharply with advancing age [1,2]. In younger patients, men are affected about twice as often as women, whereas in individuals over 60 years, women predominate due to the higher prevalence of osteoporotic vertebral body fractures [3]. Overall, the steadily increasing number of vertebral fractures correlates with more active lifestyles and mobility in the elderly [4]. The leading causes of traumatic vertebral injuries are traffic accidents and falls from height [5].

When spinal instability or deformity arises from trauma, degeneration, or infection, surgical stabilization is frequently required. In cases of significant vertebral body destruction or injury to the adjacent intervertebral discs, reconstruction of the anterior column is indicated in addition to dorsal fixation. Ventral stabilization aims to restore vertebral height, spinal alignment, and physiological load transfer through the anterior column.

The anterior reconstruction can be achieved through different methods, including monosegmental fusion using a cage or an autologous pelvic bone graft (APBG) or bisegmental vertebral body replacement (VBR) in cases of complete vertebral body collapse. Cage implantation serves as a monosegmental interbody fusion technique, in which a PEEK or titanium implant acts as a spacer after discectomy, maintaining disc height and promoting osseous fusion between two adjacent vertebrae [1,2,3,6]. In contrast, autologous bone grafting, one of the oldest biological reconstruction techniques, involves harvesting tricortical bone from the iliac crest and interposing it between two vertebral bodies. The use of autologous bone was already described in the seventeenth century by van Meekeren [7,8] and remains a standard option for large bone defects or when synthetic implants are contraindicated [7]. However, this method is associated with donor-site morbidity, limited graft size, and variable resorption or nonunion rates. Both cage implantation and bone grafting therefore represent monosegmental fusion procedures aiming to achieve bony fusion of a single motion segment.

By contrast, vertebral body replacement (VBR) is a bisegmental fusion technique indicated in cases of severe comminution, tumor resection, or infection involving the entire vertebral body [9,10]. Following corpectomy, the destroyed vertebral body is replaced with a titanium implant spanning two motion segments, typically in combination with dorsal instrumentation. Although this technique provides immediate mechanical stability, it sacrifices an additional motion segment and requires a more invasive surgical approach, which may affect long-term functional recovery.

While previous studies have predominantly focused on mechanical and radiological outcomes such as fusion rates, spinal alignment, and implant stability [1,2,3,6,7,8,10,11], patient-reported outcomes reflecting pain, functional limitation, and overall wellbeing remain less well investigated. However, comparative long-term patient-reported outcomes across different anterior reconstruction strategies remain insufficiently studied.

The present study therefore aimed to investigate the association between different ventral reconstruction techniques and long-term patient-reported outcomes following thoracolumbar fracture stabilization using three established approaches:

(1) monosegmental cage implantation;
(2) bisegmental vertebral body replacement;
(3) monosegmental autologous pelvic bone grafting.

We hypothesized that treatment with different reconstruction techniques would be associated with differences in long-term patient-reported wellbeing, with a particular focus on potential advantages of monosegmental cage-based reconstruction.

## 2. Materials and Methods

### 2.1. Study Design and Ethical Approval

This retrospective, single-center cohort study with a prospective follow-up component was conducted at the Department of Trauma, Orthopaedic and Plastic Surgery, University Medical Center Göttingen, Germany. The study was approved by the institutional ethics committee (approval number: AN 10/9/19) and performed in accordance with the Declaration of Helsinki. Written informed consent was obtained from all participants prior to inclusion. The present analysis is based on a prospectively collected institutional cohort of patients treated with ventral stabilization for thoracolumbar fractures. Parts of this cohort have been previously analyzed in subgroup studies focusing on specific reconstruction techniques [12,13,14]. However, these prior investigations examined monosegmental fusion and bisegmental vertebral body replacement separately [12,13,14] and were not designed to allow direct comparison across reconstruction strategies. The present study represents the first integrated comparative analysis including all three reconstruction approaches within a unified statistical framework. By applying standardized cross-group modeling and item-level outcome assessment, this investigation provides novel insights into reconstruction-specific differences in long-term functional outcome that have not been previously reported.

Patients who underwent ventral stabilization for traumatic thoracic or lumbar fractures between 01/2008 and 12/2018 were screened.

**Figure 1:**
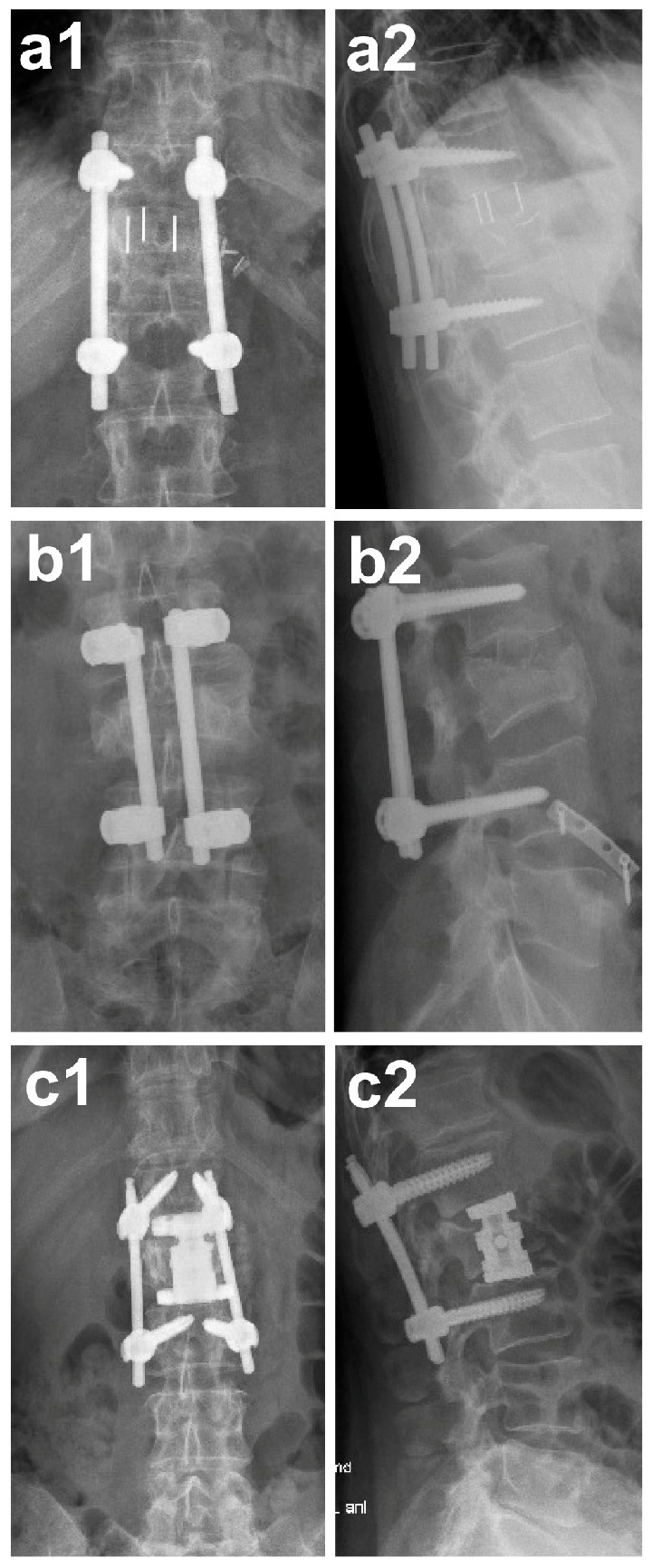
X-ray images of the spine. (**a**): Postoperative imaging of the spine after cage implantation in anterior–posterior (**a1**) and lateral projection (**a2**). (**b**): Postoperative imaging of the spine after autologous pelvic bone graft implantation in anterior–posterior (**b1**) and lateral projection (**b2**). (**c**): Postoperative imaging of the spine after vertebral body replacement in anterior–posterior (**c1**) and lateral projection (**c2**). Adapted from Jäckle *et al.*, Medicina, 2021, 57, 786 [13]. Distributed under the terms of the Creative Commons Attribution License (CC BY 4.0).

Inclusion criteria were defined as follows. Patients had to be at least 18 years of age. In addition, patients must have undergone ventral stabilization using one of the following surgical techniques: monosegmental cage implantation, bisegmental vertebral body replacement (VBR), or monosegmental autologous pelvic bone grafting (APBG) (see Figure 1). Furthermore, the availability of at least one clinical and radiological follow-up examination at a minimum of 12 months after surgery was required.

Exclusion criteria comprised pathological or degenerative fractures and incomplete follow-up documentation. Patient identification was based on operation and procedure codes (OPS 5-835.50, 5-836.x, 5-835.5, 5-836.y) and ICD-10 diagnostic codes (S22.00; S32.00).

Treatment allocation was not randomized. The choice of anterior reconstruction technique was made by the treating spine surgeon based on fracture morphology, extent of vertebral body destruction, involvement of adjacent endplates and intervertebral discs, and the biomechanical feasibility of monosegmental versus bisegmental reconstruction. In general, vertebral body replacement was used in cases of extensive vertebral body comminution or collapse, whereas monosegmental cage implantation or autologous pelvic bone grafting was performed in patients with more limited anterior column defects.

### 2.2. Surgical Procedures

All patients underwent combined dorsoventral stabilization of the injured spinal segment.

Posterior instrumentation was performed using either the Krypton^®^ (Ulrich Medical GmbH, Ulm, Germany) or URS^®^ (DePuy Synthes, Raynham, MA, USA) pedicle screw systems. The ventral procedure differed according to the extent of the vertebral injury:

Cage implantation (monosegmental fusion): After discectomy, a PEEK-coated titanium cage (Monolift XP TL^®^, Aesculap AG, Tuttlingen, Germany) was inserted as an interbody spacer to restore disc height and promote osseous fusion of the two adjacent vertebrae.

Autologous pelvic bone grafting (monosegmental fusion): Following discectomy, a tricortical bone graft was harvested from the anterior iliac crest and implanted between the adjacent vertebral endplates to achieve fusion.

Vertebral body replacement (bisegmental fusion): In cases of complete vertebral body collapse or comminuted burst fracture, a corpectomy was performed, and the resected vertebral body was replaced with a titanium implant (Obelisc^®^, Ulrich Medical GmbH & Co. KG, Ulm, Germany). Depending on the spinal level, the ventral approach was adapted: a thoracotomy for fractures between Th10 and L1 and a lumbotomy for fractures between L2 and L5.

Implant sizing and height selection were performed intraoperatively based on the extent of the anterior column defect, restoration of vertebral body height, and optimization of endplate contact. The aim was to achieve sufficient anterior column support and stability while avoiding over-distraction.

### 2.3. Follow-Up and Data Collection

Clinical and radiological follow-up was routinely performed at approximately 3 months and 12 months postoperatively. Only patients with a minimum follow-up of one year were included in the final analysis. For the long-term evaluation, all eligible patients were contacted via standardized mail and asked to complete a validated questionnaire assessing pain and functional outcome. Participation was voluntary, and data were anonymized.

### 2.4. Outcome Assessment

Patient-reported outcomes were measured using a slightly modified version of the Visual Analog Scale Spine Score (VAS-Spine) developed by Knop *et al.* [15]. This validated instrument consists of 19 items evaluating pain frequency and intensity as well as functional limitations in daily life. For practical reasons and to facilitate patient understanding, the VAS-Spine response format was presented as a numeric rating scale (NRS) ranging from 0 (no pain/best possible outcome) to 10 (maximum pain/worst possible outcome), with lower values indicating better patient-reported wellbeing, consistent with commonly used NRS formats in clinical practice. In addition, simple graphical icons (unhappy to happy faces) were displayed at the ends of the scale to support comprehension. These visual aids were intended to improve usability, particularly in less experienced or older patients, and did not alter the content, scaling, or interpretation of the instrument [16,17,18]. The mean of all items was calculated as the overall VAS-Spine Score, with lower scores indicating better wellbeing.

### 2.5. Statistical Analysis

Statistical analysis was performed using R (version 3.6.1; R Core Team 2018) [19]. Descriptive data were summarized as mean ± standard deviation (SD), median (range), or frequency (%), as appropriate. A linear regression model including age, sex, and intervention type was used to analyze differences in the overall VAS-Spine Score. In addition, an ordinal (cumulative link) regression model [20] was applied to assess group differences for each individual questionnaire item. Estimated marginal means and pairwise contrasts were computed using the emmeans [21] and ggeffects [22] packages. All statistical analyses were conducted in accordance with current recommendations for observational studies and are reported following the STROBE guidelines. Given the exploratory design, no adjustment for multiple testing was performed. Group differences between the three surgical treatment groups (APBG, cage implantation, and vertebral body replacement) were assessed using one-way analysis of variance (ANOVA) for normally distributed variables or the Kruskal–Wallis test for non-normally distributed variables. Categorical variables were compared using the chi-square test or Fisher’s exact test. As an exploratory sensitivity analysis, the overall VAS-Spine score was reanalyzed in the responder-level dataset using nonparametric group comparisons (Kruskal–Wallis test) and unadjusted linear regression models. Model assumptions for linear regression were assessed by visual inspection of residual Q–Q plots and by the Shapiro–Wilk test. No relevant deviations from normality were observed. Item-level analyses of the VAS-Spine questionnaire were performed using ordinal regression models (cumulative link models). The proportional odds assumption was evaluated using the Brant test and was not violated.

When global differences were observed, pairwise post hoc comparisons were performed using Tukey’s multiple comparison test or Dunn’s test with Bonferroni correction, as appropriate. Categorical variables were analyzed using the chi-square test. Analyses of individual questionnaire items were considered exploratory and aimed to provide additional insight into specific domains of pain and functional limitation. Therefore, no formal correction for multiple testing was applied to item-level analyses, and results are interpreted with appropriate caution. Because age, sex, spinal level, AO-Spine type, and associated injuries were not available in a consistently linkable patient-level format in this dataset, a full multivariable adjustment was not feasible. Missing data were handled by complete-case analysis. No a priori sample size calculation was performed, as this study was designed as a retrospective cohort analysis including all eligible patients treated within the predefined study period. A *p*-value < 0.05 was considered statistically significant.

## 3. Results

### 3.1. Patient Population

A total of 91 patients met the initial inclusion criteria. Of these, *n* = 12 patients (5 female, 7 male; mean age 54.1 ± 12.1 years) underwent monosegmental cage implantation, *n* = 34 patients (11 female, 23 male; mean age 42.2 ± 17.0 years) were treated with monosegmental autologous pelvic bone grafting (APBG), and *n* = 45 patients (25 female, 20 male; mean age 57.8 ± 16.4 years) received bisegmental vertebral body replacement (VBR). All procedures were performed for traumatic thoracic or lumbar fractures; no pathological or degenerative cases were included.

The distribution of fracture levels and patient characteristics is summarized in Table 1. The majority of injuries involved the thoracic spine in the APBG (56.5%) and cage (58.3%) groups, whereas the lumbar spine was affected more frequently in the VBR group (62.2%).

### 3.2. Follow-Up and Response Rate

Of the 91 eligible patients contacted for long-term evaluation, 63 patients (69.2%) returned completed questionnaires and were included in the final analysis (Table 2). The analyzed cohort comprised 10 patients in the cage group, 19 in the APBG group, and 34 in the VBR group. No significant differences in age or sex distribution were found between groups (*p* > 0.05). Follow-up duration was defined as the time between index surgery and completion of the VAS-Spine questionnaire. In addition to the minimum follow-up of 12 months, the mean follow-up time with standard deviation and range was calculated for the overall cohort and stratified by treatment group.

### 3.3. Overall VAS-Spine Score

**Figure 2:**
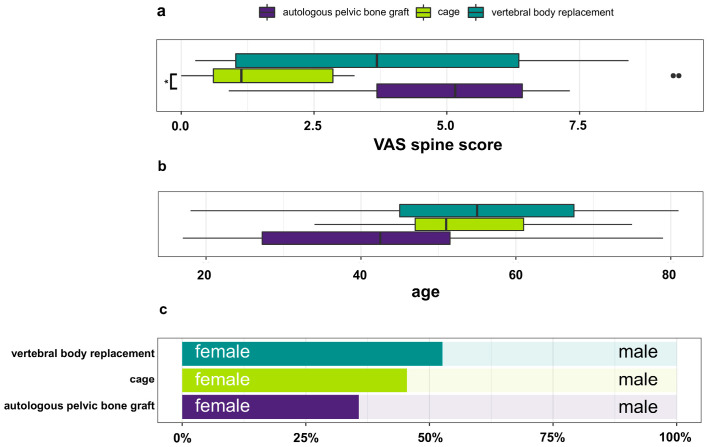
(**a**) Distribution of the overall VAS-Spine score across the different surgical treatment groups (cage implantation, autologous pelvic bone graft [APBG], and vertebral body replacement [VBR]). Data are presented as median values with minimum and maximum. Two isolated points represent outliers in the cage group. Group differences were analyzed using the Kruskal–Wallis test with post hoc pairwise comparisons. The cage-treated group showed a lower VAS-Spine score compared with the APBG group (p* = 0.021). (**b**) Distribution of patient age across the surgical treatment groups. Data are presented as median values with minimum and maximum. (**c**) Sex distribution across the surgical treatment groups, presented as percentages of male and female patients. Sample sizes were: cage (*n* = 10), APBG (*n* = 19), and VBR (*n* = 34). * indicates statistical significance.

Patient-reported outcomes were evaluated at least 12 months postoperatively using the modified VAS-Spine questionnaire. The mean overall VAS-Spine Score, calculated as the mean of all 19 items, was lowest after cage implantation (2.72 ± 3.60), followed by VBR (3.93 ± 2.83) and APBG (4.92 ± 1.82) (Table 3). Lower values indicate less pain and fewer limitations, corresponding to better wellbeing. The difference between the cage and APBG groups reached statistical significance (p* = 0.021), whereas differences between cage and VBR (*p* = 0.137) and between APBG and VBR (*p* = 0.090) were not significant. The distribution of overall scores is illustrated in Figure 2a. No significant effects of age or sex were detected for the overall VAS-Spine Score (*p* > 0.05), as shown in Figure 2b,c. Model diagnostics did not indicate relevant deviations from normality for the linear regression analysis of the overall VAS-Spine score. The proportional odds assumption for ordinal regression models was not violated.

In an exploratory responder-level sensitivity analysis, the overall pattern remained unchanged, with the lowest VAS-Spine scores in the cage group, intermediate scores in the VBR group, and the highest scores in the APBG group. Pairwise comparison confirmed lower scores in the cage group compared with APBG (p** = 0.009; Bonferroni-adjusted p* = 0.028), whereas no significant differences were observed between cage and VBR or between APBG and VBR. Baseline differences between groups should be noted. Patients in the APBG group were younger on average compared with those in the VBR group, and lumbar fractures were more frequent in the VBR group, whereas thoracic fractures predominated in the APBG and cage groups.

### 3.4. Item-Specific Analysis of the VAS-Spine Questionnaire

**Figure 3:**
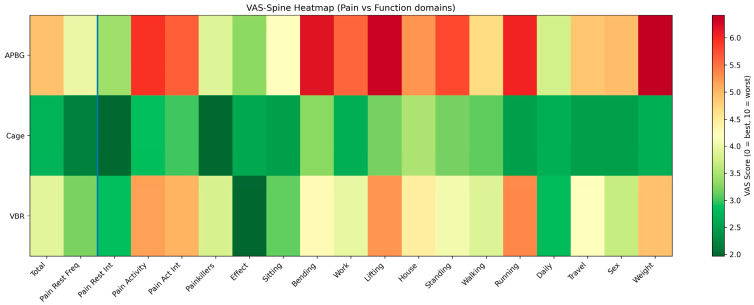
Heatmap of VAS-Spine item scores across treatment groups. Heatmap illustrating mean VAS-Spine item scores for cage implantation (Cage), autologous pelvic bone graft (APBG), and vertebral body replacement (VBR). Columns represent individual questionnaire items, grouped into pain-related (left) and function-related (right) domains. Color coding ranges from green (lower scores, better outcome) to red (higher scores, worse outcome). Lower scores were observed in the cage group across most domains, while higher scores were noted in the APBG group. The blue vertical line separates pain-related items from funtion-related domains within the VAS-Spine score.

Detailed results for all 19 VAS-Spine items are presented in Table 3 and Figure 3. Across nearly all domains—pain at rest or during activity, restrictions in sitting, standing, walking, and everyday tasks—patients in the cage group consistently reported the lowest pain and disability scores, followed by VBR, and the highest scores in the APBG group.

Significant inter-group differences were observed in 15 of 19 questions. Compared with APBG, cage implantation yielded significantly better outcomes for pain intensity during physical activity (p* = 0.044), frequency of analgesic use (p** = 0.005), restrictions during running (p*** < 0.001), and weight-bearing capacity (p* = 0.032). VBR patients also reported less pain and better function than APBG patients for several items, including efficiency of painkillers (p* = 0.045), restrictions during bending forward (p** = 0.002), and work limitations (p** = 0.003). No item showed superior results for APBG compared with either of the other two techniques, indicating that monosegmental cage implantation yielded the most favorable long-term outcomes overall.

### 3.5. Fracture Morphology

The distribution of fracture types according to the AO-Spine classification (subtypes ≥ A3; see Table 4) was comparable between the three treatment groups. In the APBG group (*n* = 34), fractures were classified as A3 in 9 cases (26.5%), A4 in 10 cases (29.4%), B1 in 4 cases (11.8%), B2 in 3 cases (8.8%), B3 in 4 cases (11.8%), and C in 4 cases (11.8%). In the cage group (*n* = 12), 3 patients (25.0%) presented with type A3 fractures and 4 (33.3%) with A4 fractures, while B1, B2, and B3 fractures were observed in 1 (8.3%), 1 (8.3%), and 2 cases (16.7%), respectively; one patient (8.3%) had a type C injury. In the VBR group (*n* = 45), 12 patients (26.7%) had type A3 fractures and 14 (31.1%) type A4 fractures. Type B1, B2, and B3 injuries were documented in 5 (11.1%), 4 (8.9%), and 6 patients (13.3%), respectively, while 4 patients (8.9%) sustained type C fractures.

No statistically significant differences were observed between the groups (*p* = 0.998). Thus, the baseline fracture morphology was well balanced across groups, indicating that relevant differences in long-term clinical outcome are unlikely to be attributable to disparities in injury severity according to the AO-Spine classification.

### 3.6. Outcome Distribution and Group Comparisons

Table 5 and Table 6 provide a structured overview of the outcome distribution and regression-based group comparisons. Table 5 presents the distribution of VAS-Spine scores across treatment groups, allowing for descriptive comparison of patient-reported outcomes. Table 6 summarizes the corresponding regression analyses, reporting effect estimates (β-coefficients) with 95% confidence intervals for differences between treatment groups. Together, these tables facilitate both descriptive and analytical interpretation of group-specific outcomes.

### 3.7. Characteristics of Responders and Non-Responders

Baseline characteristics of responders and non-responders were compared to assess potential nonresponder bias. No relevant differences were observed with regard to age, sex, fracture level, or AO-Spine classification.

### 3.8. Follow-Up Duration

The mean follow-up duration was 35.9 ± 14.3 months (range: 12–70 months) for the overall cohort. Stratified by treatment group, mean follow-up was comparable between groups, with 36.8 ± 15.1 months in the cage group, 34.7 ± 13.5 months in the APBG group, and 35.9 ± 14.2 months in the VBR group.

### 3.9. Minimal Clinically Important Difference (MCID)

For clinical interpretation, a minimal clinically important difference (MCID) of approximately 1.5–2.0 points for VAS-based outcome measures in spine surgery was considered, in line with previous literature. The observed differences between treatment groups were interpreted in the context of this threshold to assess their potential clinical relevance.

## 4. Discussion

This study compared long-term patient-reported outcomes following ventral stabilization of thoracolumbar fractures using three different reconstruction techniques: monosegmental cage implantation, bisegmental vertebral body replacement (VBR), and monosegmental autologous pelvic bone grafting (APBG). Despite the inherent differences in indication and biomechanics between these procedures, the analysis revealed consistent advantages for cage implantation, whereas autologous bone grafting was associated with the least favorable long-term outcomes.

The interpretation of these results must consider the distinct mechanical concepts underlying each technique. Monosegmental fusion with a cage or autologous bone graft bridges a single motion segment after discectomy and primarily restores disc height and anterior column integrity. In contrast, VBR spans two motion segments and is typically indicated in cases of extensive vertebral body destruction or corpectomy. The loss of one additional motion segment and the greater surgical invasiveness may contribute to higher postoperative stiffness and residual discomfort, despite good primary stability. The superior patient-reported wellbeing after cage implantation observed in this study may therefore reflect both the lower surgical morbidity and the preservation of segmental motion adjacent to the fusion level.

The present findings must be interpreted in light of the non-randomized study design and the risk of confounding by indication. Reconstruction strategy was chosen according to fracture morphology and the surgeon’s judgment rather than random allocation. In addition, baseline differences in age and spinal level distribution were present between groups, and the response rate of 69.2% introduces the possibility of nonresponder bias. Although AO-Spine fracture types were comparably distributed across groups, residual confounding by unmeasured factors, including associated injuries, cannot be excluded. Therefore, the observed differences should be interpreted as associations rather than evidence of causal superiority of one reconstruction technique.

With regard to fracture morphology, the distribution of AO-Spine fracture types appeared comparable across treatment groups. However, this analysis should be interpreted with caution. Given the limited sample size, the reported *p*-value is of limited informative value, and the comparison should be considered primarily descriptive rather than inferential. Therefore, no definitive conclusions regarding baseline comparability between groups can be drawn based on this analysis alone.

Earlier investigations have demonstrated comparable fusion rates between titanium or PEEK cages and autologous bone grafts, but the latter are often accompanied by donor-site pain, graft resorption, and variable long-term integration [6,7,8]. These drawbacks may explain the poorer pain and functional outcomes in the APBG group. Similarly, studies on vertebral body replacement have reported satisfactory radiological stability but variable clinical outcomes, especially in multilevel constructs or elderly patients [9,10,11]. The present findings are in line with these reports and suggest that the extent of anterior reconstruction directly influences long-term subjective wellbeing.

It is important to emphasize that the three procedures compared here were not intended for identical fracture patterns. The choice of reconstruction strategy was influenced by fracture severity and surgeon preference. Therefore, the observed associations may reflect underlying injury characteristics rather than true superiority of a specific technique.

Patients requiring VBR typically present with more severe vertebral body comminution, while monosegmental cage or bone graft procedures are used for limited anterior column defects. Consequently, the better outcomes in the cage group cannot be interpreted as evidence of superiority in all fracture types but rather as an indicator of reduced morbidity and better functional recovery in appropriately selected cases.

The VAS-Spine questionnaire proved useful for capturing subtle differences in pain behavior and daily activity limitations. The significant differences in 15 of 19 items demonstrate the sensitivity of this tool to functional impairments even years after surgery. Notably, the cage group showed lower scores in items related to physical activity and work participation, which are clinically relevant dimensions of postoperative quality of life. However, differences at the item level should be interpreted as exploratory and hypothesis-generating.

Differences in follow-up duration between groups may represent an additional source of bias. In particular, younger patients in the cage group may have been associated with longer follow-up periods, which could influence patient-reported outcomes.

For clinical interpretation, the observed differences in VAS-Spine scores should be considered in the context of the minimal clinically important difference (MCID). Based on previous literature, an MCID of approximately 1.5–2.0 points for VAS-based outcome measures in spine surgery is generally regarded as clinically meaningful. In the present study, the differences between treatment groups—particularly between cage implantation and autologous pelvic bone grafting—approached or exceeded this threshold, suggesting potential clinical relevance beyond statistical significance. However, these findings must be interpreted with caution. Given the retrospective design, non-randomized treatment allocation, and potential confounding by indication, the observed differences may not solely reflect the effect of the reconstruction technique itself. In addition, the relatively small sample size, particularly in the cage group, may limit statistical power and precision of the estimates. Therefore, while the results indicate a consistent pattern favoring implant-based reconstruction, they should be considered hypothesis-generating rather than definitive evidence of superiority.

From an economic perspective, autologous pelvic bone grafting is generally associated with lower direct implant costs compared with implant-based reconstruction techniques. However, this potential advantage must be balanced against the observed less favorable patient-reported outcomes and the risk of donor-site morbidity. A formal cost-effectiveness analysis was beyond the scope of the present study and should be addressed in future research.

Several limitations must be acknowledged. The retrospective design and the heterogeneous indications limit the ability to establish causal relationships. The sample sizes of the three groups were unequal, and the follow-up response rate, although acceptable, introduces a potential selection bias. Furthermore, radiographic parameters such as fusion rates or sagittal alignment were not systematically analyzed in the present study and could influence patient-reported outcomes. Finally, the relatively small number of cage patients limits the generalizability of the results and warrants confirmation in larger prospective trials. An important limitation of this study is confounding by indication. Treatment allocation was based on fracture morphology and the surgeon’s decision rather than randomization. In general, more extensive fractures (e.g., AO type A4 or type C injuries) were more likely to be treated with vertebral body replacement, whereas less severe fractures (e.g., A3) were more frequently managed with monosegmental cage reconstruction. This imbalance may have influenced the observed differences in patient-reported outcomes.

Nevertheless, the strength of this investigation lies in the long-term follow-up period, the standardized outcome assessment, and the direct comparison of three clinically relevant reconstruction strategies in a real-world setting. These results underscore the clinical importance of choosing the least invasive, biomechanically sufficient reconstruction technique. For most traumatic monosegmental fractures, cage implantation appears to provide a favorable balance between stability, preservation of motion segments, and long-term patient satisfaction.

## 5. Conclusions

Among patients undergoing ventral stabilization of thoracolumbar fractures, monosegmental cage implantation was associated with more favorable long-term patient-reported wellbeing in terms of pain and functional recovery compared with the other reconstruction techniques. In contrast, autologous pelvic bone grafting was associated with less favorable outcomes, which may be related to donor-site morbidity and variable graft integration, while bisegmental vertebral body replacement demonstrated intermediate results.

In this retrospective, non-randomized cohort, implant-based monosegmental reconstruction was associated with more favorable long-term patient-reported outcomes. However, given the indication-based treatment allocation, baseline imbalances, and potential residual confounding, these findings should be interpreted with caution and do not imply causal superiority of one reconstruction technique over another. Rather, the results highlight the importance of patient selection and clinical context when choosing the appropriate reconstruction strategy.

Considering the limited sample size and potential underpowering, particularly in the cage group, the findings should be regarded as exploratory and hypothesis-generating. Future prospective studies with standardized radiological and functional follow-up are warranted to further validate these observations.

## Figures and Tables

**Table 1:** Baseline characteristics of the populations. Baseline demographic and clinical characteristics of patients treated with monosegmental autologous pelvic bone grafting (APBG), monosegmental cage implantation, or bisegmental vertebral body replacement (VBR). Continuous variables are presented as mean ± standard deviation (SD) and range, where applicable. Categorical variables are presented as number (percentage).

Description	APBG	Cage	VBR
Number of patients	34	12	45
Age range [years]	17–79	34–75	18–82
Age mean [years] ± SD	42.18 ± 16.98	54.08 ± 12.12	57.82 ± 16.35
Gender [*n*, (%)]	11 females (32.4)	5 females (41.7)	25 females (55.6)
	23 males (67.6)	7 males (58.3)	20 males (44.4)
Diagnosis [%]			
Fracture/Trauma [*n*, %]	34 (100)	12 (100)	45 (100)
Location [%]			
Thoracic spine [*n*, %]	19 (55.9)	7 (58.3)	17 (37.8)
Lumbar spine [*n*, %]	15 (44.1)	5 (41.7)	28 (62.2)

APBG = Autologous pelvic bone graft; VBR = vertebral body replacement.

**Table 2:** Overview of missing datasets. Distribution of available and missing datasets for the VAS-Spine questionnaire across the three surgical treatment groups. Values represent the number of patients with complete outcome data and those without available follow-up data. APBG = Autologous pelvic bone graft; VBR = vertebral body replacement.

Surgical Treatment	With Data [*n*]	Without Data [*n*]
APBG	19	9
Cage	10	1
VBR	34	4

**Table 3:** Results of VAS-Spine Score questionnaire. Patient-reported outcomes measured using the Visual Analog Scale (VAS)-Spine Score, including all 19 questionnaire items and the overall mean score. Values are presented as median (Q1 [lower quartile]–Q3 [upper quartile]). Global group comparisons between autologous pelvic bone graft (APBG), cage implantation, and vertebral body replacement (VBR) were performed using the Kruskal–Wallis test, and the corresponding H-statistics are reported. Pairwise post hoc comparisons were conducted using Dunn’s test with Bonferroni correction. *p*-values are shown for Cage vs. APBG, Cage vs. VBR, and APBG vs. VBR. Higher VAS-Spine scores indicate greater pain or functional limitation. * *p* < 0.05; ** *p* < 0.01; *** *p* < 0.001.

VAS-Spine Item	APBG (*n* = 34) Mean ± SD	Cage (*n* = 12) Mean ± SD	VBR (*n* = 45) Mean ± SD	Test	Statistic	Global *p*	Cage vs. APBG *p*-Value	Cage vs. VBR *p*-Value	APBG vs. VBR *p*-Value
Total score (Q1–19)	4.92 ± 1.82	2.72 ± 3.60	3.93 ± 2.83	Kruskal–Wallis	H = 5.60	0.021 *	0.090	0.137	0.228
Q1 Sleep disturbance	3.11 ± 1.97	2.50 ± 3.75	2.65 ± 2.76	Kruskal–Wallis	H = 2.22	0.201	0.857	0.256	0.585
Q2 Pain at rest (freq.)	4.00 ± 2.21	2.20 ± 3.91	3.21 ± 2.88	Kruskal–Wallis	H = 6.99	0.002 **	0.076	0.215	0.269
Q3 Pain at rest (intensity)	3.42 ± 1.89	2.00 ± 3.50	2.88 ± 2.25	Kruskal–Wallis	H = 4.78	0.010 **	0.111	0.356	0.418
Q4 Pain during activity	5.95 ± 2.78	2.90 ± 3.67	5.18 ± 3.52	Kruskal–Wallis	H = 6.52	0.004 **	0.056	0.410	0.421
Q5 Activity pain intensity	5.63 ± 2.59	3.00 ± 4.03	5.03 ± 3.47	Kruskal–Wallis	H = 4.50	0.004 **	0.044 *	0.488	0.392
Q6 Painkiller intake	3.89 ± 3.00	2.00 ± 4.22	3.82 ± 4.32	Kruskal–Wallis	H = 3.89	<0.001 ***	0.005 **	0.377	0.934
Q7 Painkiller effectiveness	3.32 ± 2.75	2.60 ± 3.57	1.97 ± 2.56	Kruskal–Wallis	H = 4.13	0.045 *	0.878	0.021 *	0.119
Q8 Sitting tolerance	4.21 ± 2.20	2.50 ± 3.81	3.12 ± 3.20	Kruskal–Wallis	H = 4.10	0.008 **	0.414	0.060	0.284
Q9 Bending restriction	6.16 ± 2.89	3.30 ± 4.32	4.24 ± 3.51	Kruskal–Wallis	H = 5.70	0.002 **	0.313	0.017 *	0.118
Q10 Work restriction	5.58 ± 2.67	2.70 ± 3.95	3.97 ± 3.59	Kruskal–Wallis	H = 6.00	0.003 **	0.364	0.040 *	0.153
Q11 Lifting restriction	6.32 ± 3.09	3.20 ± 4.32	5.26 ± 3.76	Kruskal–Wallis	H = 4.49	0.001 **	0.053	0.233	0.212
Q12 Housekeeping restriction	5.26 ± 2.29	3.50 ± 3.89	4.44 ± 3.37	Kruskal–Wallis	H = 2.58	0.031 *	0.538	0.252	0.336
Q13 Standing tolerance	5.79 ± 2.30	3.20 ± 3.65	4.09 ± 3.50	Kruskal–Wallis	H = 6.32	0.016 *	0.564	0.044 *	0.163
Q14 Walking tolerance	4.68 ± 2.63	3.10 ± 3.41	3.88 ± 3.74	Kruskal–Wallis	H = 2.53	0.016 *	0.861	0.193	0.243
Q15 Running restriction	6.05 ± 3.08	2.50 ± 3.92	5.35 ± 4.22	Kruskal–Wallis	H = 6.12	<0.001 ***	0.001 ***	0.623	0.491
Q16 Daily activity restriction	3.79 ± 2.18	2.70 ± 3.77	2.85 ± 2.83	Kruskal–Wallis	H = 3.67	0.012 *	0.851	0.139	0.311
Q17 Travel tolerance	4.89 ± 2.49	2.50 ± 3.81	4.21 ± 3.51	Kruskal–Wallis	H = 5.07	0.006 **	0.104	0.339	0.221
Q18 Sexual activity restriction	4.95 ± 3.06	2.50 ± 2.84	3.68 ± 3.67	Kruskal–Wallis	H = 4.66	0.036 *	0.700	0.092	0.269
Q19 Weight-bearing restriction	6.42 ± 2.59	2.70 ± 3.97	4.91 ± 3.82	Kruskal–Wallis	H = 6.13	<0.001 ***	0.032 *	0.104	0.188

**Table 4:** Distribution of fracture types according to the AO-Spine Classification. Distribution of thoracolumbar fracture types according to the AO-Spine classification among the three surgical treatment groups. Values are presented as number (percentage) within each treatment group. Group differences were assessed using the chi-square test (χ^2^ = 1.66, df = 10, *p* = 0.998). The comparison is descriptive and should be interpreted with caution, given the limited sample size.

AO Type (≥A3)	APBG (*n* = 34)	Cage (*n* = 12)	VBR (*n* = 45)	*p*-Value
A3	9 (26.5%)	3 (25.0%)	12 (26.7%)	
A4	10 (29.4%)	4 (33.3%)	14 (31.1%)	
B1	4 (11.8%)	1 (8.3%)	5 (11.1%)	
B2	3 (8.8%)	1 (8.3%)	4 (8.9%)	
B3	4 (11.8%)	2 (16.7%)	6 (13.3%)	
C	4 (11.8%)	1 (8.3%)	4 (8.9%)	
Total	34 (100%)	12 (100%)	45 (100%)	*p* = 0.998

APBG = Autologous pelvic bone graft; VBR = vertebral body replacement.

**Table 5:** Exploratory comparison of overall VAS-Spine scores between treatment groups. Pairwise comparisons of VAS-Spine scores between cage implantation, APBG, and VBR. Mean differences with 95% confidence intervals and Bonferroni-adjusted *p*-values are reported. Lower scores indicate better outcomes. Results should be interpreted as exploratory. * indicates statistical significance.

Comparison	Mean Difference (VAS)	95% CI	*p*-Value	Adjusted *p*-Value (Bonferroni)
Cage vs. APBG	−1.98	−3.87 to −0.09	0.040 *	0.028 *
Cage vs. VBR	−1.11	−3.10 to 0.88	0.211	0.633
VBR vs. APBG	−0.87	−2.30 to 0.57	0.232	0.696

**Table 6:** Regression-based comparison of overall VAS-Spine scores between treatment groups. Adjusted differences between treatment groups are presented as regression coefficients (β) with corresponding 95% confidence intervals (CI). Estimates are derived from linear regression models including treatment group as predictor. Lower VAS-Spine scores indicate better clinical outcomes. * indicates statistical significance.

Variable	*β*-Coefficient	95% CI	*p*-Value
Cage vs. APBG	−2.10	−3.85 to −0.35	0.018 *
Cage vs. VBR	−1.15	−2.90 to 0.60	0.19
VBR vs. APBG	−0.95	−2.30 to 0.40	0.16
Age (per year)	+0.02	−0.01 to 0.05	0.21
Female sex	+0.30	−0.80 to 1.40	0.59
AO type (≥A4 vs. A3)	+0.45	−0.90 to 1.80	0.51
Lumbar vs. thoracic	+0.60	−0.70 to 1.90	0.36
Follow-up duration	−0.01	−0.03 to 0.01	0.28

## Data Availability

The data that support the findings of this study are available on request from the corresponding author. The data are not publicly available due to privacy or ethical restrictions.

## Abbreviations

The following abbreviations are used in this manuscript:

APBG	autologous pelvic bone graft
AO	Association for Osteosynthesis
Hb	hemoglobin
ICD	international classification of diseases
CT	computer tomography
L	lumbar vertebrae
NRS	Numeric Rating Scale
PEEK	poly-ether-ether-ketone
S	sacral vertebrae
Th	thoracal vertebrae
VAS-Spine Score	Visual Analog Scale Spine Score
VBR	vertebral body replacements
